# Ethics and Professional Influence

**Published:** 1913-02

**Authors:** 


### Ethics and Professional Influence

An editorial is supposed to present the ultimate wisdom on a point of scientific fact or professional policy. Unfortunately, there are editors, who realize the importance of certain moot points but do not possess the wisdom to pronounce a final verdict. Sometimes the editor realizes this lack, sometimes he does not. The present is one of the cases in which it seems best to present an unsolved problem.

We have always been in hearty accord with and have endeavored to practice and uphold the principles and rules of medical ethics. Even medical etiquette has seemed more important in many instances than the name implies. The only exceptions have been in a few instances in which a codified rule exaggerates the importance of an arbitrary opinion or a minor principle of ethics should be subordinated to a principle so fundamental that it will ordinarily be considered one of common honesty and morals, rather than ethics.

In intercourse with members of other professions, and especially with business men, we have been impressed with the very general opinion that the medical profession is warped in its views, fussy about certain ideals, narrow in its outlook, and, if not too selfish, at least too hide-bound, to be trusted to regulate the various problems of professional polity and to control others, as it seeks to do by its influence on popular and legislative opinion.

This is most unfortunate, for the medical profession is charitable, if anything it is too indifferent to its own material welfare, rarely or never does even a small body of medical men attempt to exert an influence to secure any law which it does not consider disinterestedly, and the profession, more than any other, has consistently sacrificed its own interests to those of the community.

Do we need to climb to a higher viewpoint to secure a broader conception of ethical problems? If so, how shall we attain it? If not, how shall we dispel the unfavorable opinion of our critics?

